# Geography of COVID-19 in Denmark

**DOI:** 10.1177/1403494820975607

**Published:** 2020-11-25

**Authors:** Therese Lf Holmager, Elsebeth Lynge, Caroline E Kann, Gry St-Martin

**Affiliations:** Centre for Epidemiological Research, Nykøbing Falster Hospital, University of Copenhagen, Denmark

**Keywords:** COVID-19, Denmark, population characteristics, health planning, mortality, hospitalization

## Abstract

*Aim:* To investigate the COVID-19 situation across geographical areas of Denmark over time. *Methods:* We used COVID-19 data from the Danish State Serum Institute on national, regional and municipality level. Cumulative number of tests, incidence, hospitalizations and deaths per 100,000 inhabitants were analysed for the five Danish regions and for all of Denmark. The cumulative number of tested and incidence of COVID-19 per 100,000 was compared for the two municipalities, Lolland and Gentofte. A sensitivity analysis of the COVID-19 indicators on a regional level was performed using number of tested as the denominator. *Results:* The Capital Region ranked highest on all analysed COVID-19 indicators with 10,849 tested, 365 cases, 63 hospitalized and 18 deaths per 100,000 by 2 June 2020. The three regions in western Denmark all had low levels, while Region Zealand ranked second highest. Despite general low health status in Lolland municipality, the cumulative incidence of COVID-19 was consistently below that of Gentofte. Sensitivity analysis showed that the Capital Region had the highest number of COVID-19 cases per 100,000 tested, but Region Zealand had a higher number of hospitalized and similar number of deaths per 100,000 tested as the Capital Region over time. ***Conclusion*: COVID-19 had affected eastern Denmark, especially the Capital Region, considerably more than western Denmark. The difference may relate to population density and housing conditions.**

## Introduction

The first known case of COVID-19 in Denmark was diagnosed on 27 February 2020 [1]. The first patients returned from skiing holidays in Italy and Austria and generated local chains of transmission. For two weeks, the health authorities aimed to contain the infection through isolation of cases and contact tracing. However, by 10 March, the majority of cases were acquired in Denmark and the strategy changed from containment to mitigation [1,2]. The prime minister announced a temporary closedown of schools and childcare institutions. Public employees without critical functions were required to work from home [1]. Hospitals prepared for an inflow of COVID-19 patients and outpatient visits and elective surgery were postponed [3]. Private businesses with person-to-person contact, for example restaurants, entertainment venues, hairdressers and many shops, closed down temporarily and gatherings of more than 10 persons were banned [1,2]. From mid-April, a gradual reopening of society started with childcare and schools, and by 2 June most businesses, restaurants, etc. had reopened with limited capacity due to distancing measures [4].

As SARS-CoV-2 is a new pathogen, all persons are potentially susceptible, but disease manifestations vary considerably, from asymptomatic cases to fatal disease. Children usually develop mild or no symptoms, although severe manifestations, for example suspected Kawasaki disease or toxic shock syndrome [5], have been described. Elderly, frail people, on the other hand, are at high risk of developing severe, life threatening disease [6]. In adults, the risk of severe disease depends on underlying health status. Obesity [7] and chronic diseases including diabetes, respiratory or cardiovascular disease increase the risk for severe COVID-19 [6].

Denmark is a small country, considered to have good and fairly equal health status [8]. Nevertheless, health disparities are found between the five regions and 98 municipalities, with life expectancy in the rural, provincial municipality of Lolland almost six years behind that of rich Gentofte north of Copenhagen [9]. Region Zealand has the lowest life expectancy with 80.5 years while in Central Region, it is 81.7 years [9]. In the National Health Surveys, Region Zealand reported a high prevalence of daily smoking, obesity and diabetes compared with the other regions [10].

With this background of known regional differences in risk factors for severe COVID-19, we hypothesized that Region Zealand would experience higher rates of hospitalization and death from COVID-19 than the other regions. We investigated the incidence of COVID-19, COVID-19 related hospitalizations and deaths across the five regions in Denmark.

## Material and methods

### Data

We compared COVID-19 indicators across the five Danish regions; Capital Region of Denmark (Capital), Region Zealand (Zealand), Region of Southern Denmark (South), Central Denmark Region (Central) and North Denmark Region (North). Capital and Zealand together form the eastern part of Denmark, while the remaining three regions form the western part. Furthermore, the two municipalities Gentofte (in Capital) and Lolland (in Zealand) with the highest and lowest life expectancy were compared.

We retrieved COVID-19 data from COVID-19 surveillance reports from the State Serum Institute (SSI) up to 2 June 2020 [11], including cumulated number of cases (from 16 March by region and 27 March by municipality), cumulated number of tested persons (from 16 April by region and 17 April by municipality), cumulated number of deaths and hospitalizations (from 16 April by region). SSI retrieved the number of persons tested and number testing positive from the Danish Microbiology Database (MiBa), and hospitalized patients with COVID-19 from the Danish National Patient Registry (LPR3) and daily reports from regions. Deaths with COVID-19 were identified from the Danish Central Population Register (CPR) and the Cause of Death Register [12]. SSI used personal identification numbers to link individuals across registers, identify municipality of residence, and to delete duplicates.

In SSI reports, a person testing for COVID-19 was defined as a person having a throat swap sample submitted for SARS-CoV-2 testing at a regional clinical microbiology laboratory, SSI’s lab, tested through the TestCenter DK [12]. All samples and results were registered in MiBa. A COVID-19 case was defined as a person testing positive for COVID-19 antigen and were registered by sampling date. Hospitalization with COVID-19 was defined as a patient hospitalized for at least 12 hours or any length of time in intensive care during the 14 days following sampling date of a positive SARS-CoV-2 test or as a patient testing positive during hospitalization [12]. A death with COVID-19 was defined as a death occurring ⩽ 30 days after a positive SARS-CoV-2 test, regardless of reported cause of death [12].

We retrieved data on population size, age distribution and housing from StatBank Denmark [9], and area size from Danish Regions [13].

### Recommendations for testing

National recommendations for testing were updated several times. Testing activity further depended on the availability of test equipment and the set-up of test facilities, especially during the first two months. The main test recommendations being [12]:

- Until 12 March 2020, testing of symptomatic travellers returning from high-risk areas, hospitalized patients with symptoms of COVID-19, and contacts of known cases. Persons with mild symptoms were not tested but encouraged to self-isolate.
- From 12 March to 1 April 2020, mainly patients hospitalized for suspected COVID-19 were tested. Contact tracing was abandoned.
- From 1 April 2020 onwards, patients with mild to moderate symptoms, both hospitalized or seen in general practice, and healthcare and nursing home staff with mild symptoms and close contact to patients were also tested.
- From 21 April 2020 onwards, testing of all symptomatic persons, regardless of severity, and asymptomatic persons including all hospitalized patients and in certain cases staff in hospitals, nursing homes and residential facilities, was recommended.
- From 18 May 2020, all adults could book a COVID-19 antigen test without medical referral. Contact tracing and testing were again encouraged [14].

### Statistical analysis

We analysed the development in four indicators across regions: (a) cumulative number of people tested for COVID-19 per 100,000; (b) cumulative incidence of COVID-19 per 100,000; (c) cumulative hospitalization rate per 100,000; and (d) cumulative mortality rate with COVID-19 per 100,000. Gentofte and Lolland municipalities were compared using indicators 1 and 2.

As only persons tested for COVID-19 could contribute to indicators 2, 3 and 4, it was supplemented with an analysis using the cumulative number of COVID-19 tested persons as denominator.

## Results

### Population data

The Danish population included 5.8 million inhabitants, ranging from 590,000 in the North to 1.8 million in Capital (Table I). Proportion of inhabitants ⩾ 70 years ranged from 13% in Capital to 17% in Zealand, while the proportion ⩾ 80 years was 4–5% in all regions. Population density was 721 people per km^2^ in Capital, while ranging from 115 to 74 in other regions [13]. In Capital, 56% lived in apartments, v. 20–26% in other regions [9].

**Table I.** Characteristics and COVID-19 indicators for the five Danish regions and total Denmark, 2 June 2020.

	Capital	Zealand	South	Central	North	Total
Population in 1000	1846	837	1223	1326	590	5823
% of population aged ⩾ 70 years	13	17	16	14	16	14
% of population aged ⩾ 80 years	4	5	5	4	5	5
Region area (km^2^)	2561	7273	12191	13142	7931	43,098
People per (km^2^)	721	115	100	101	74	135
Housing (%)						
House	44	80	78	73	78	66
Apartment	56	20	22	26	22	34
One-person households	19	17	18	17	19	18
COVID-19 tested	200,268	76,358	99,308	104,669	52,491	533,094
COVID-19 positive	6737	1887	1005	1596	437	11662
Hospitalized with COVID-19	1169	477	245	290	101	2282
Dead with COVID-19	337	118	34	69	22	580
No. tested positive, hospitalized (%)	17	25	24	18	23	20
No. tested, hospitalized (%)	0.58	0.62	0.25	0.28	0.19	0.43
Deaths as % of positive	5	6	3	4	5	5
Deaths as % of tested	0.17	0.15	0.03	0.07	0.04	0.11

### Status by 2 June 2020

By 2 June 2020, 533,094 inhabitants had been tested for COVID-19 of whom 11,662 (2.2%) tested positive, equivalent to 200 COVID-19 cases per 100,000, of whom 2282 (19.6%) had been hospitalized and 580 (5.0%) died, corresponding to 39 hospitalizations and 10 deaths per 100,000.

### Test activity

**Figure 1.**
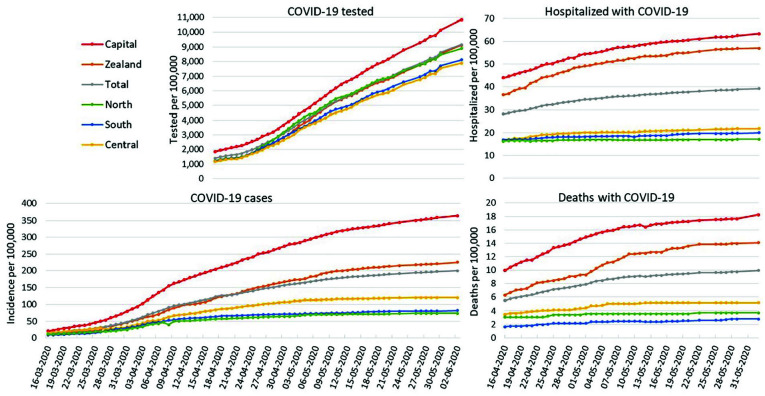
Cumulated incidence of COVID-19 (from 16 March to 2 June 2020), cumulative number of COVID-19 tested persons, cumulative number of persons hospitalized with COVID-19 and cumulative number of deaths with COVID-19 (from 16 April to 2 June 2020) per 100,000 inhabitants, for the five Danish regions and total Denmark. Date is day–month–year. On several days, there was a decrease in the cumulated incidence of COVID-19 due to corrections of registration errors over time.

Proportion of inhabitants tested was close to the national average in Zealand and North, higher in Capital and lower in South and Central. In Capital, more than 10% were tested, while this was 8% in Central and South (Figure 1). For all regions, the increase in cumulative proportion of inhabitants tested was steeper from around 22 April 2020.

### Cumulative incidence

Capital had the highest cumulative incidence by 2 June 2020 at 365 per 100,000, compared to 225 in Zealand and 74–120 in western regions. When number of tested was used as denominator, Capital and Zealand still had higher cumulative incidence than the western regions (Figure 1).

**Figure 2.**
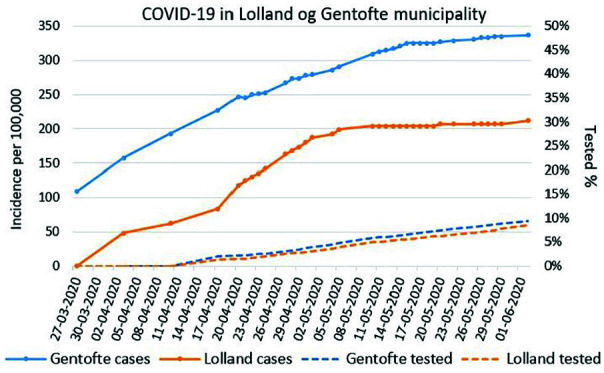
Cumulated incidence of COVID-19 (from 27 March to 2 June 2020) per 100,000 inhabitants and cumulative percentage of COVID-19 tested persons (from 17 April to 2 June 2020) for Gentofte and Lolland municipalities. Date is day–month–year. On several days, there was a decrease in the cumulated incidence of COVID-19 due to corrections of registration errors over time. Lolland municipality had < 10 cases (below the reporting limit) on 27 March.

A slightly higher proportion of inhabitants was tested in Gentofte than in Lolland, 9.4 and 8.5%, respectively (Figure 2), but the cumulative incidence was considerably higher in Gentofte than in Lolland, 337 and 121 cases per 100,000, respectively.

### Hospitalization and deaths

**Figure 3.**
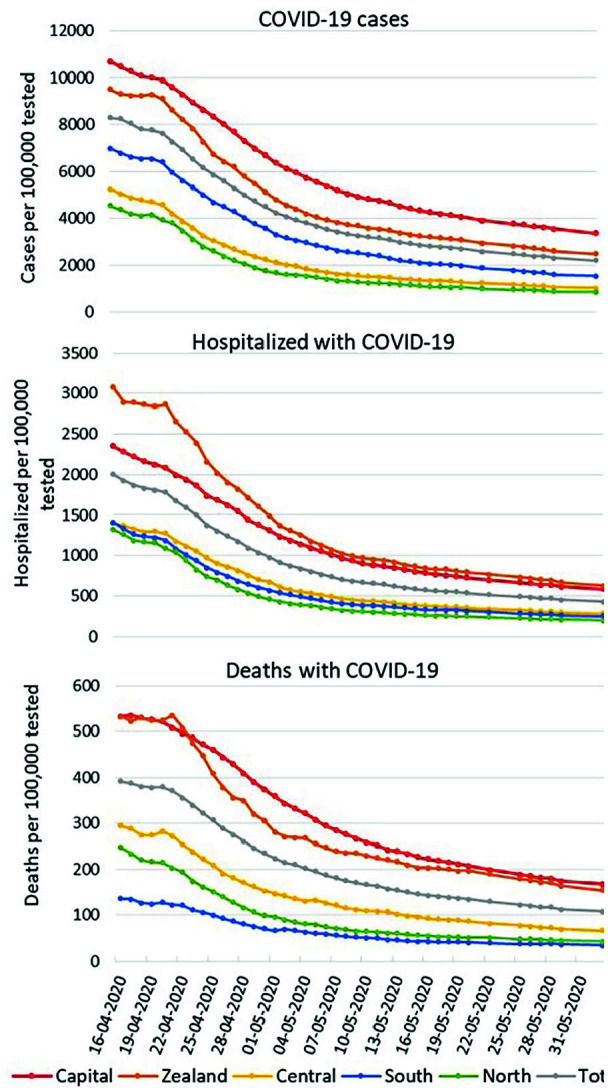
Cumulated number of COVID-19 cases, cumulative number of persons hospitalized with COVID-19 and cumulative number of deaths with COVID-19 per 100,000 COVID-19 tested persons for the five Danish regions and total Denmark from 16 April to 2 June 2020. Date is day–month–year. On several days, there was a decrease in the cumulated incidence of COVID-19 due to corrections of registration errors over time.

Until 2 June 2020, the cumulative hospitalization rate with COVID-19 was highest for Capital, 63 per 100,000, followed by Zealand, 57 per 100,000. Western regions had lower rates; 17–22 per 100,000 (Figure 1). However, hospitalization rate calculated with number of tested as denominator for Zealand was above that of Capital until beginning of May, thereafter the rates aligned (Figure 3).

Likewise, Capital had the highest cumulative mortality rate with COVID-19, by 2 June 2020 18 deaths per 100,000 in the Capital, 14 in Zealand and 5, 4 and 3 per 100,000, in the remaining regions (Figure 1). Mortality rates calculated with number of tested as denominator were 533 per 100,000 tested for both Capital and Zealand on 16 April 2020 and decreased from 22 April 2020, as number of tested increased (Figure 3). Until 2 May, Zealand decreased faster than Capital but onwards rates aligned.

## Discussion

### Main finding

We hypothesized that Zealand would experience more COVID-19 hospitalizations and deaths than other regions because inhabitants in this region were more vulnerable than the average Danish inhabitant and would, if infected, be more prone to severe disease.

The data did not confirm our hypothesis. Throughout the three and a half months studied, Capital had the highest cumulative incidence, hospitalization and mortality rates. This difference was seen even when comparing Lolland and Gentofte municipalities, the two extremes in terms of life expectancy. Rates in Zealand were below Capital, but considerably higher than the three western regions of Denmark. However, until early May, the hospitalization rate per tested inhabitants was higher in Zealand than in the Capital, even though the Capital had more positive cases per tested inhabitants than Zealand.

### Other Nordic countries

Among the Nordic countries by 2 June 2020, Sweden had the highest cumulative incidence of COVID-19 with 370 per 100,000 and the highest death rate of 43 per 100,000 [15]. Norway and Finland had lower cumulative incidence and death rates for COVID-19 than Denmark. Finland had the lowest cumulative incidence, 125 per 100,000, and Norway had the lowest cumulative death rate, 4 per 100,000 [15].

At the beginning of March, when COVID-19 started to spread in Sweden, it mainly affected the capital Stockholm and Jämtland Härjedalen (the region of Sweden’s largest skiing resorts) [16]. Differences in the cumulative incidence of COVID-19 were seen across the 21 counties; from 611 per 100,000 in the central county, Örebro, and 582 in Stockholm, to 133 in the most southern county, Skåne, and low incidence in the rural counties Götland, Kalmar, Norrbotton and Västerbotten [16]. Geographical variation in cumulative incidence of COVID-19 was seen also in Norway from 381 per 100,000 in the capital county, Oslo, to 50 in the northern county, Nordland [17]. In Finland, the incidence in the capital region was much higher than in other regions, and the Finnish Government introduced a temporary ban on travel to and from the capital region from 28 March to 15 April 2020 [18,19].

The Swedish government implemented fewer societal measures than the other Nordic countries and, for example, did not close shops, schools or borders [20]. In addition, Sweden had severe outbreaks in care homes for older people, which contributed to the high death rate [20]. Unlike the other Nordic countries, Sweden experienced marked increases in overall mortality compared to previous years [21].

### Danish reports

Among COVID-19 cases in Denmark, 42% were men and 17% were aged ⩾ 70 years [12]. Of the COVID-19 deaths, 57% were men and 87% were ⩾ 70 years [12]. Overall, 19% of COVID-19 cases were hospitalized; 17% in women and 20% in men [12]. Hospitalization rate increased with age from less than 10% of cases age < 50 years, to over 50% at age ⩾ 70 years [12].

Comorbidity has been a strong predictor for hospitalization of COVID-19 infected persons [22], and for all age groups, 71–85% of deaths with COVID-19 occurred in patients having one or more comorbidities [12]. Proposed explanations for higher risk of severe disease among men and elderly include higher prevalence of comorbidities and delayed healthcare seeking [6,23].

As in other western countries, immigrants from non-western countries have been particularly at risk for COVID-19, constituting 19% of cases but only 9% of the population [24,25]. This was not explained by increased testing but may be linked to a high proportion of immigrants living in Capital, and many immigrants/descendants were infected at home reflecting larger households (more than five persons) [24,26].

### Strengths and limitations

The Danish nationwide registration systems allowed for rapid and reliable statistics on COVID-19 tests and results. Personal identification numbers allowed for counting of unique persons even at re-testing. Indicators for COVID-19 were linked with the person’s address, except for hospitalization which was linked to the hospital’s region [12]. For certain specialized treatments, patients from other regions are normally transferred, for example to Capital, however, during the epidemic many of these treatments were postponed to prepare for an extra inflow of COVID-19 patients. All regions established wards for treatment of patients with COVID-19, thus the vast majority of COVID-19 hospitalizations were presumed to take place in the region of residence.

It is a limitation of the study that data on age and comorbidities of cases were reported only on a national and not a regional level in the surveillance reports. However, COVID-19 hospitalization and death rates were highest in people aged ⩾ 70 years, and population statistics did not indicate that observed regional differences in COVID-19 indicators could be attributed to differences in age distribution.

Criteria for testing changed over time and testing capacity increased gradually. This limited the interpretation of data. Number of hospitalized cases were less affected by this than total number of cases, because cases severe enough to require hospitalization were always tested. However, from 22 April 2020, patients hospitalized for other causes were routinely tested for COVID-19, regardless of symptoms. If testing positive, they would meet the definition for ‘hospitalized with COVID-19’, even if the infection was not severe enough to warrant hospitalization, but no data were available to estimate to which extent this occurred. In addition, deaths from other causes than COVID-19 would be registered as a COVID-19 death, if the person tested positive ⩽ 30 days before death.

### Interpretation of findings

Our study demonstrated considerable regional differences in COVID-19 indicators in Denmark.

Importantly, our study also highlights the difficulties in the interpretation of COVID-19 surveillance data. Number of cases diagnosed depends on the interplay between number of people infected (i.e. spread of the epidemic), number of cases with symptoms or severe symptoms (partly dependent on underlying health/comorbidities and age), and on testing activity. All of these varied over time and across regions. Number of people tested depended on care-seeking behaviour, referral from doctors and self-referral for testing when this option was introduced mid-May. No data are available on the proportion of symptomatic or asymptomatic persons tested by region or whether care-seeking behaviour or self-referral for testing differed between regions. On the national level, age differences in test activity, cumulative incidence and risk for hospitalization and deaths have been demonstrated [12,22] and our study showed a higher proportion of COVID-19 positive hospitalized in the three regions with highest proportion of older people (Table I), but we do not have complete regional data to explore this further.

Nevertheless, our study indicated that the disease burden from COVID-19 was larger in Capital than in the rest of Denmark and this could not be ascribed solely to more intensive testing.

A number of factors may have contributed to the geographic pattern observed. Complying with distancing and self-isolation recommendations may have been more difficult in Capital, where the population density is about seven times higher [13] and more people use public transport than in the rest of Denmark [9]. 56% of inhabitants in Capital live in apartments as opposed to 20–26% in the rest of Denmark [9]. The high incidence in Capital was also reflected in the risk for immigrants, many of whom live in Capital. The epidemic also hit earlier in Capital: from 1 to 10 March, 30 patients had been hospitalized in Capital and Zealand, while the western regions had their first hospitalizations on 10 March (4 patients) [11].

Capital and Zealand are located on the same island, and extensive commuting for work takes place between these two regions, which may explain why Zealand, in spite of population density and housing comparable to the western regions, had an incidence closer to that of Capital. In addition, travel between the eastern and western parts of Denmark clearly dropped during the COVID-19 epidemic, limiting further spread from Zealand to the western regions. Whereas 441,000 train passengers and 1,127,030 motor vehicles crossed the Great Belt Bridge in April 2019, only 108,000 train passengers and 568,808 vehicles crossed in April 2020 [27–29].

While more cases per 100,000 were diagnosed in Capital, a higher proportion of cases were hospitalized in Zealand. This is compatible with a combination of higher test activity in Capital but a more vulnerable population (i.e. more at risk of severe disease leading to hospitalization) in Zealand, but might also reflect a local shortage of testing equipment in the early phase of the epidemic leading to more selective testing of mainly severe cases.

In summary, we hypothesize that the lower incidence in western Denmark was at least partly explained by a combination of fewer introductions of COVID-19 at the start of the epidemic, slower spread due to lower population density, less use of public transport and living primarily in single houses, perhaps complemented by lower test activity.

Our findings showed large variation in geographical spread of COVID-19 in Denmark, indicating that local strategies may be relevant for future handling of epidemics. High population density as well as large households seemed to increase the risk of COVID-19, thus, opportunities for self-isolation outside one’s home may help to reduce transmission.

## Conclusion

COVID-19 affected eastern Denmark, especially Capital, more than western Denmark. Published data on age, gender and comorbidities of cases were not available by region, but possible reasons for the higher COVID-19 burden in Capital included higher population density, dwelling in apartments, use of public transport and initially more imported cases. Risk factors for severe disease such as age ⩾ 70 years and obesity were highest in Zealand and lowest in Capital. Increased incidence of COVID-19 in capital areas was observed also in the other Nordic countries.
